# The bidirectional association among female hormone‐related cancers: breast, ovary, and uterine corpus

**DOI:** 10.1002/cam4.1473

**Published:** 2018-04-16

**Authors:** Min‐Chi Chen, Kuan‐Der Lee, Chang‐Hsien Lu, Ting‐Yao Wang, Shih‐Hao Huang, Chao‐Yu Chen

**Affiliations:** ^1^ Department of Public Health Biostatistics Consulting Center College of Medicine Chang Gung University Taoyuan Taiwan; ^2^ Department of Hematology & Oncology Chang Gung Memorial Hospital Chiayi Taiwan; ^3^ Division of Hematology and Oncology Department of Internal Medicine Taipei Medical University Hospital Taipei Taiwan; ^4^ School of Medicine College of Medicine Taipei Medical University Taipei Taiwan; ^5^ Department of Gynecology and Obstetrics Chang Gung Memorial Hospital Chiayi Taiwan

**Keywords:** Breast cancer, ovarian cancer, second cancer, Taiwan Cancer Registry, uterine cancer

## Abstract

Breast, ovarian, and uterine corpus cancers are common female cancers and categorized as hormone‐related diseases. Previous studies reported a unidirectional relationship for each cancer, but few studied the reciprocal association in the same cohort. A population‐based study was carried out in Taiwan to test the hypothesis that there are pairwise bidirectional associations among these cancers. Using the same cohort of 110,112 cases with primary female cancers including uterine corpus cancer (11,146 cases), ovarian cancer (12,139 cases), or breast cancer (86,827 cases) from the Taiwan Cancer Registry from 1979 to 2008, the pairwise risks of second cancer among uterine corpus, ovary, and breast cancer cases were evaluated by standardized incidence ratios (SIRs) and the corresponding 95% confidence intervals (CIs) to quantify the excess of second malignancies. A reciprocal relationship was found for these three female cancers, particularly most prominent between uterine and ovarian cancers, followed by breast and uterine cancers as well as breast and ovarian cancers. The overall risk of second cancers was highest within the first 5 years after the diagnosis of primary cancer. The bidirectional relationships suggest common risk factors among these three female cancers. This is the largest cohort study to focus on the bidirectional associations among hormone‐related cancers in Asian women, and these results could aid in the development of early prevention strategies and follow‐up surveillance programs.

## Introduction

Breast, uterine corpus, and ovarian cancers are common female cancers in Taiwan. According to the Taiwan Cancer Registry (TCR), the incidences of these three cancers have increased rapidly in the Taiwanese population in recent decades, ranking first, sixth, and seventh as of 2014 1, 2. Many risk factors that contribute to breast cancer are also associated with gynecological cancers, such as estrogen exposure, reproductive status, genetic factors, and obesity, which likely contribute to the higher incidences 3.

It has been known that patients with breast cancer have a higher incidence of second uterine cancers 4, 5. Tamoxifen and reproductive risks probably contribute to the higher incidence 5. BRCA1/2 gene mutations that are usually seen in triple negative breast cancer would also increase the risk of uterine cancer, especially at an early age 6, 7. However, the risk of second breast cancer after primary uterine cancer remains disputed. Win et al. 8 found a modest increase in risk of second breast cancer after endometrial cancer for women with Lynch syndrome, but other Western studies do not support this finding 9, 10, 11.

The risk of second ovarian cancer is increased in young patients with primary uterine cancer 12, 13. Hung et al. 14 found that patients with primary ovarian cancer also have an increased risk of second uterine cancer, but Levi showed that patients with ovarian cancer did not have any excess risk of any second cancer in his study that recruited 1530 subjects 15. Regarding the association between ovarian and breast cancers, it is evidenced by the increased rates of second ovarian cancer after breast cancer in BRCA1/2 mutation carriers 4, 5, 16. As to the patients without BRCA1/2 mutation, the results were inconsistent 10, 14, 15.

A unidirectional association is defined as a one‐way relationship between two cancers, where having a primary cancer increases the relative risk of subsequent cancer, but the reverse may not be true. In contrast, a bidirectional association implies a two‐way or reciprocal relationship between two cancers, regardless of which cancer occurs first 17. The bidirectional effect between uterine cancer and breast cancer was demonstrated by Cortesi et al. 18, who showed that the bidirectional risk was not exclusively due to the effect of tamoxifen. Hemminki also found the bidirectional effect between ovarian and uterine cancers and suggested that genetic effects might explain these associations 10, 18. Nevertheless, very few studies were addressed the reciprocal relationship of these female cancers in Asian populations. Thus, we conducted a population‐based study by examining the pairwise associations among uterine, ovary, and breast cancers using the same cohort population for the period of 1979–2008. Elucidation of the relationships between these three female cancers could provide important implications for understanding the etiologic mechanisms, developing follow‐up strategies, and for early detection and treatment.

## Materials and Methods

### Data sources

We quantified the risk of second cancer among patients who were diagnosed with uterine corpus, ovary, or breast cancer with results reported the results to the TCR (http://tcr.cph.ntu.edu.tw/) between 1 January 1979 and 31 December 2008. The TCR was founded in 1979 and is financed by the Ministry of Health and Welfare for estimating the incidence of cancer in Taiwan. It is a population‐based cancer registry that covered 22 million people and 97.6% of cancer patients in 2006 19. The quality of TCR database was assured by two quality indexes: (1) the DCO% (percent of death certificate only cases) for all cancers ranges between 1.24% and 2.34%. It is as low as 0.25–0.67% and 0.07–0.33% for breast cancer and uterine corpus cancer, respectively, and (2) the overall MV% (percent of morphological verification) ranges between 90% and 92% (Cancer Registry Annual Report, 2004–2008). All cancer registry databases in the TCR have been systemically converted to codes from the International Classification of Diseases, 9th Revision 20, and linked with death certificates that were last updated in December 2009 from the National Death Database.

People not identified by this process were considered to be alive for the purposes of this study (passive follow‐up). The coding of multiple primaries followed the principles of the International Association of Cancer Registries (IACR) and the International Agency for Research on Cancer (IARC) 21, 22. Informed consent was not required because all registry records were anonymous and open to the public.

This analytical cohort included 115,171 women who were diagnosed at age >20 years with uterine corpus, ovary, or breast cancer from 1979 to 2008. To minimize any potentially unconfirmed cancer diagnoses and to estimate the person‐year follow‐up, we excluded 5059 patients from analysis because they met at least one of the following criteria: (1) missing birth dates, last follow‐up date, or death status (1215 cases); or (2) second cancer diagnosis or death occurring <2 months after the primary cancer (3881 cases). As a result, a total of 110,112 cases were included in the analysis. Among them, 11,146 patients had primary uterine corpus cancer (ICD‐9: 182), 12,139 had ovarian cancer (ICD‐9: 183), and 86,827 had breast cancer (ICD‐9: 174).

### Statistical analysis

The standardized incidence ratios (SIRs) and the corresponding 95% confidence intervals (CIs) were calculated to quantify the excess of second malignancies after the diagnosis of primary cancer 23. SIRs were taken as the ratio of the observed number (O) of second cancers to the expected number (E), which was obtained by assuming that these people experienced the same cancer incidence as the corresponding general female population. The number of person‐years at risk was defined as the number of years from the date of initial diagnosis of the primary cancer to the date of death, date of last follow‐up, date of the diagnosis of second primary cancers, or the end of the study period (December 31, 2008), whichever came first. The person‐years of observation for 5‐year age groups and 5‐year periods were multiplied by the incidence rates of cancers for the Taiwanese female population. The corresponding products were summed over all ages and calendar years to yield the expected number of second cancers at each site. The confidence intervals of SIRs were based on the assumption of a Poisson distribution of second cancer cases.

The Kaplan–Meier survival curves were used to examine the survival time after primary cancers and after second cancers. The overall survival was compared using the log rank test, and the differences among curves after second cancers are presented by the hazard ratio using the Cox proportional hazards model, in which age at onset (<50 years vs. ≥50 years) was adjusted. All statistical tests were two‐sided, and *P* < 0.05 was considered statistically significant.

## Results

### Patient characteristics

In this cohort, there were 110,112 cases with primary female cancers including uterine corpus cancer (11,146 cases), ovary cancer (12,139 cases), or breast cancer (86,827 cases). Among them, 3515 (3.19%) developed at least one‐second primary malignancy (SPM) during 663,102 person‐years of follow‐up. In these 3515 SPM cases, 596 cases (17.06%) were second female cancers (Table 1). Overall, the average follow‐up time was 6.02 years, including 49,726 cases (45%) followed up for at least 5 years and 21,916 cases (20%) for over 10 years.

**Table 1:** Characteristics of population‐based cohort of 110,112 patients with primary diagnosis of uterine corpus, ovary, or breast cancer in Taiwan, 1979–2008

First primary cancer site	Uterine corpus (ICD 182)	Ovary (ICD 183)	Breast (ICD 174)
No. with first primary cancer	11,146	12,139	86,827
Average age at diagnosis of first primary cancer ±SD (years)	52.97 ± 11.58	50.52 ± 14.95	51.10 ± 12.16
No. who developed a second cancer (%)^a^	598 (5.37%)	436 (3.59%)	2481 (2.86%)
Second uterine corpus (%)^b^		28 (6.42%)	238 (9.59%)
Ovary (%)^b^	28 (4.68%)		140 (5.64%)
Breast (%)^b^	90 (15.05%)	72 (15.81%)	
Others (%)^b^	480 (80.27%)	336 (77.06%)	2149 (84.76%)
Average follow‐up (years)	5.94 ± 5.35	5.49 ± 5.45	6.11 ± 5.18

SD, standard deviation; ICD, International Classification of Diseases, 9th Revision.

a Percentage of all patients with first primary cancer.

b Percentage of patients with any second cancers.

### Bidirectional risk of second cancer

The pairwise risks of second cancer among uterine corpus, ovary, and breast cancer cases were evaluated by SIRs and corresponding 95% CIs (Table 2). The risk excess between two pairwise female cancers was increased reciprocally, suggesting that patients who had primary uterine, ovary, or breast cancer were at a higher risk of developing a second cancer in female organs than the general female population. In these female cancers, the strongest bidirectional association was found between ovary and uterine corpus cancer (SIR = 3.26, 95% CI = 2.17–4.71, and SIR = 3.04, 95% CI = 2.02–4.40), followed by breast and uterine cancer (SIR = 2.96, 95% CI = 2.60–3.37, and SIR = 1.41, 95% CI = 1.14–1.74), and breast and ovary cancer (SIR = 1.99, 95% CI = 1.67–2.35, and SIR = 1.32, 95% CI = 1.03–1.66).

**Table 2:** Bidirectional risk among uterine, ovary, and breast cancers

Second primary	First primary		
	Uterine corpus	Ovary	Breast
	SIR O/E (95% CI)	SIR O/E (95% CI)	SIR O/E (95% CI)
Uterine corpus		**3.26** * 28/8.58 (2.17–4.71)	**2.96** * 238/80.30 (2.60–3.37)
Ovary	**3.04** * 28/9.20 (2.02–4.40)		**1.99** * 140/70.44 (1.67–2.35)
Breast	**1.41** * 90/63.41 (1.14–1.74)	**1.32** * 72/54.51 (1.03–1.66)	

Three entries in each cell are SIR, O/E, and 95% CI, where SIR = standardized incidence ratios; CI = confidence interval; O = observed number of second cancers; E = expected number of second cancers.

Bold SIR with * indicates statistical significance (*P* <0.05).

### Bidirectional risk stratified by age at initial diagnosis

To examine the effect of menopause, the SIRs were stratified by the age at initial diagnosis of the primary cancer (<50 and ≥50 years; Table 3). For primary uterine cancer, the risk of second ovary was significantly higher in those <50 years old, but the risk of second breast cancer was higher for those ≥50 years old. For patients with primary ovarian cancer, the risk of second uterine and breast cancer was only seen in those <50 years old. For primary breast cancer, the risk of second ovarian cancer was significantly higher in those <50 years old, but the risk of second uterine cancer was higher for those ≥50 years old. Menopause seems to have an effect on the bidirectional risk. For young patients <50 years old, a strong bidirectional association was observed between uterine and ovarian cancers (SIR = 5.27, 95% CI = 3.11–8.32, and SIR = 4.78, 95% CI = 2.92–7.38), and for those after 50 years of age, the trend was strongest between breast and uterine cancers.

**Table 3:** Bidirectional risk among uterine, ovary, and breast cancers stratified by age at diagnosis of first primary cancer

Second primary	First primary cancer					
	Uterine		Ovary		Breast	
	<50 yr	≥50 yr	<50 yr	≥50 yr	<50 yr	≥50 yr
	SIR O/E (95% CI)	SIR O/E (95% CI)	SIR O/E (95% CI)	SIR O/E (95% CI)	SIR O/E (95% CI)	SIR O/E (95% CI)
Uterine			**4.78** * 20/4.18 (2.92–7.38)	1.82 8/4.40 (0.78–3.58)	**2.16** * 88/40.68 (1.73–2.66)	**3.79** * 150/39.61 (3.20–4.44)
Ovary	**5.27** * 18/3.42 (3.11–8.32)	1.73 10/5.78 (0.83–3.18)			**2.48** * 85/34.29 (1.98–3.07)	**1.52** * 55/36.16 (1.14–1.98)
Breast	1.13 31/27.34 (0.77–1.61)	**1.64** * 59/36.07 (1.25–2.11)	**1.42** * 42/29.66 (1.02–1.91)	1.21 30/24.85 (0.81–1.72)		

Three entries in each cell are SIR, O/E, and 95% CI, where SIR = standardized incidence ratios; CI = confidence interval; O = observed number of second cancers; E = expected number of second cancers. yr= years old.

Bold SIR with * indicates statistical significance (*P* <0.05).

### Bidirectional risk stratified by follow‐up interval after primary cancer

To explore the latency of occurrence of second cancers, the SIRs were stratified by time interval since the diagnosis of primary cancer (Table 4). The entire follow‐up period was categorized into three intervals: ≤5 years, 5–10 years, and >10 years. Intriguingly, for patients with primary uterine and ovarian cancers, the risk of developing second female cancers was only increased during the first 5 years of follow‐up only. In contrast, patients with primary breast cancer showed a lifetime risk of second uterine cancer and a 10–year risk of second ovarian cancer. During the first 5 years of follow‐up, uterine and ovarian cancers showed a strong bidirectional excess of risk (SIR = 13.78, 95% CI = 8.92–20.34; SIR = 11.83, 95% CI = 7.65–17.46).

**Table 4:** Bidirectional risk among uterine, ovary, and breast cancers stratified by follow‐up after diagnosis of first cancer

Follow‐up interval (yr)	1st Uterine cancer	
	2nd Ovary	2nd Breast
≤5	**13.78** * 25/1.81 **(8.92**–**20.34**)	**3.62** * 49/13.54 (2.68–4.78)
5–10	0.36 1/2.77 (0.00–2.01)	1.05 21/19.95 (0.65–1.61)
>10	0.44 2/4.60 (0.05–1.57)	0.67 20/29.92 (0.41–1.03)

	1st Ovarian cancer	
	2nd Uterine	2nd Breast
≤5	**11.83** * 25/2.11 (7.65–17.46)	**2.87** * 37/12.90 (2.02–3.95)
5–10	0.81 2/2.46 (0.09–2.93)	1.30 20/15.42 (0.79–2.00)
>10	0.25 1/4.01(0–1.39)	0.57 15/26.19 (0.32–0.94)

	1st Breast cancer	
	2nd Uterine	2nd Ovary
≤5	**5.03** * 86/17.10 (4.02–6.21)	**4.51** * 63/13.98 (3.46–5.76)
5–10	**3.14** * 84/26.71 (2.51–3.89)	**1.76** * 40/22.72(1.26–2.40)
>10	**1.86** * 68/36.49 (1.45–2.36)	1.10 37/33.74 (0.77–1.51)

Three entries in each cell are SIR, O/E, and 95% CI, where SIR = standardized incidence ratios; CI = confidence interval; O = observed number of second cancers; E = expected number of second cancers. yr = years.

Bold SIR with * indicates statistical significance (*P* <0.05).

### Overall survival of the primary and second cancers

**Figure 1:**
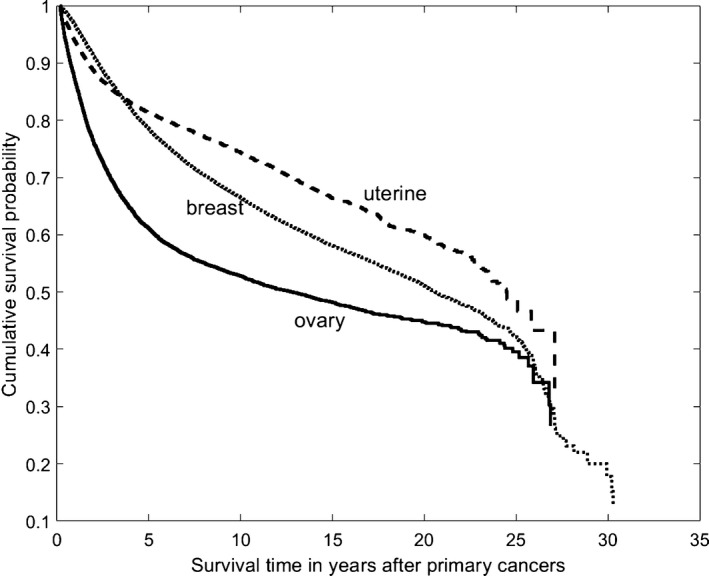
Kaplan–Meier survival curves of patients with primary uterine corpus, breast, or ovarian cancer.

The overall median survival time was 20.80 years for all of these three female cancers. For stratification by the primary tumor site, the median survival times were 24.46, 20.59, and 12.77 years for primary uterine, breast and ovarian cancers, with 5‐year survival rates of 81%, 79%, and 61%, respectively (Table 5). The survival rates were significantly different from each other (*P* < 0.001 in all cases), in which uterine cancer was associated with the best survival, followed by breast cancer in comparison with ovarian cancer (Fig. 1).

**Table 5:** Overall survival and survival probability after developing second cancers

1st primary cancer		Survival probability					Median survival (years)
		1 year	5 years	10 years	15 years	20 years	
Uterine	All (*n* = 11,146)	0.94	0.81	0.74	0.66	0.60	24.46
	No. of patients at risk	10,448	6519	3177	1380	458	
	w/SPM (*n* = 598)	0.74	0.47	0.39	0.318	0.26	4.00
	2nd ovary	0.89	0.60	0.51	0.32	0.32	10.14
	2nd breast	0.96	0.68	0.56	0.56	0	19.39
	Others	0.69	0.43	0.36	0.28	0.25	2.97
Ovary	All (*n* = 12,139)	0.87	0.61	0.53	0.48	0.45	12.77
	No. of patients at risk	10,537	5787	3098	1463	535	
	w/SPM (*n* = 436)	0.70	0.40	0.30	0.27	0.24	2.73
	2nd uterine	0.89	0.67	0.60	0.60	0.60	NA
	2nd breast	0.90	0.59	0.37	0.37	0.37	8.48
	Others	0.64	0.34	0.26	0.22	0.18	1.90
Breast	All (*n* = 86,827)	0.97	0.79	0.67	0.58	0.51	20.59
	No. of patients at risk	83,813	51,227	24,128	9914	3384	
	w/SPM (*n* = 2481)	0.75	0.44	0.33	0.28	0.22	3.81
	2nd uterine	0.85	0.56	0.51	0.49	0.36	10.49
	2nd ovary	0.86	0.45	0.41	0.33	0.33	4.36
	Others	0.73	0.42	0.30	0.26	0.20	3.12

w/SPM, with second primary malignancy.

**Figure 2:**
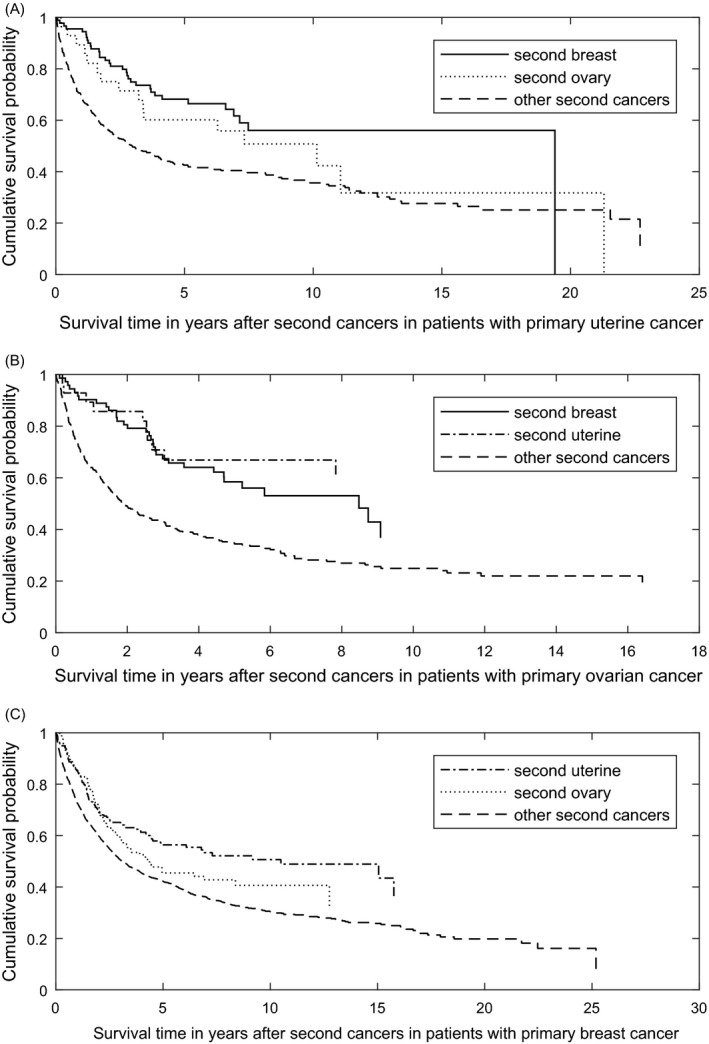
Survival curves of (A) second breast and ovarian cancers for patients with primary uterine corpus cancer; (B) second uterine corpus and breast cancers for patients with primary ovarian cancer; and (C) second uterine corpus and ovarian cancers for patients with primary breast cancer.

The survivorship after second cancer was related to the second tumor site. For primary uterine cancer, second breast cancer has a better survival than second ovarian cancer and other second cancers (HR = 0.54 with *P* = 0.048 and HR = 0.49 with *P* < 0.001, respectively) (Fig. 2A). For primary ovarian cancer, both second uterine and breast cancers were associated with a better survival than other second cancers (HR = 0.367 with *P* = 0.002, and HR = 0.510 with *P* < 0.001, respectively) (Fig. 2B). For primary breast cancer, second uterine cancer survived longer than second ovarian cancer and other cancer sites (HR = 0.74 with *P* = 0.047 and HR = 0.628 with *P* < 0.001, respectively), and there was no difference between second ovary and others (HR = 0.85 with *P* = 0.166) (Fig. 2C).

## Discussion

Similar to other Asian countries, the incidences of breast, uterine, and ovarian cancers in Taiwan have been increasing in the recent decades 2. Due to the evolution of treatment and early detection by cancer screening, the survival of these patients has been prolonged 10, 18, 24. However, the incidence of second cancer has also increased as a result. We have shown that patients with breast, uterine, or ovarian cancers are at higher risk of subsequently developing second cancer than the general population. A reciprocal relationship was found for these three female cancers, particularly more prominent between uterine and ovarian cancers, as well as ovarian and breast cancers.

Unidirectional associations are more likely to be due to cancer treatment effects. However, bidirectional associations tend to be implied by shared genes, environment, and intrinsic and extrinsic hormone exposure 17. Breast cancer shares common risk factors for gynecologic cancers, such as estrogen exposure, BRCA1/2 genes, obesity, early menarche, late menopause, low parity, and infertility 3, 25. Patients with primary uterine cancer, especially postmenopausal women (≥50 years), have an increased risk of second breast cancers. This could be explained in part by the lifestyle changes caused by the rapid industrialization that has occurred in Taiwan. A more Westernized lifestyle could have resulted in more patients with metabolic syndrome, which includes obesity, type 2 diabetes, and dyslipidemia. Metabolic syndrome increases the risk of endometrial and breast cancers in postmenopausal women 26.

Genetic components, particularly genes involved in estrogen synthesis or metabolism, may play a role in the emergence of estrogen‐related malignancies in young Asian women 2. Young age at onset, racial differences, Lynch syndrome, and BRCA1/2 mutation all suggest that genetic factors are important 9, 10, 27. For instance, the incidence of second breast cancer in ovarian cancer patients with BRAC1/2 mutation was reported to be 8.9% 16, and the risk of second uterine cancer is also significant in BRCA1 carriers (SIR = 1.91, *P* = 0.03), but not in BRCA2 carriers (SIR = 1.75, *P* = 0.20) 6. Hall et al. found that young women with uterine cancer have higher incidences of second ovarian cancer that can be attributed to genetic factors 12, 28. Despite small sample size, the prevalence of germline BRCA1/2 mutations was similar in Taiwan compared with Western countries in cases of ovarian cancer (26.1% in serous carcinoma) or breast cancer (11.1% in early‐onset, or familial breast cancer) 29, 30.

In our study, the bidirectional relationship between breast and uterine cancers was prominent in patients with age ≥50 years old. Estrogen receptor‐positive breast cancer patients treated with tamoxifen are known to have an elevated risk of subsequent uterine cancer 31. Notably, patients with invasive breast cancer have a higher risk of developing subsequent uterine cancer, regardless of hormone receptor status 7. Mellemkjær et al. 4 found that higher risk was noted before 1975 when tamoxifen was rarely used. The increased risk of uterine cancers for hormone receptor‐negative breast cancer survivors raises concerns about other factors in addition to tamoxifen 7.

Another major finding in this study is that the highest risk of developing second cancers was seen in the first 5 years. Pertaining to survival, second ovarian cancer had the worst outcome, in which could be explained by ovarian cancer generally being diagnosed at an advanced stage and often having a poor outcome 32. Therefore, a close surveillance for mammography and gynecological ultrasound examination should be advocated for those patients with primary endometrial, ovarian, or breast cancer especially in the first 5 years.

In conclusion, our study represents the first population‐based study in Asian women enlightening a reciprocal relationship between hormone‐related cancers by their risk of developing a second cancer. However, a major limitation in our study is the lack of some information on potential confounders. Our study does not include information about parity, lifestyle, body mass index, treatment modality, use of oral contraceptives, and staging. Hence, we could not examine how these factors relate to the risk of developing second cancers. For instance, some literature suggests that the use of oral contraceptives and hormone‐related cancers are associated. However, we could not evaluate this association in our study. The major strength of our study is the nationwide, long‐term follow‐up of survival status, and the size of the cohort, which is large and homogeneous. The results thus provide a more accurate estimation of the incidence and risk of the bidirectional association between breast, uterine, and ovarian cancers.

## Conflict of Interest

None declared.
